# Non-Viral Generation of Marmoset Monkey iPS Cells by a Six-Factor-in-One-Vector Approach

**DOI:** 10.1371/journal.pone.0118424

**Published:** 2015-03-18

**Authors:** Katharina Debowski, Rita Warthemann, Jana Lentes, Gabriela Salinas-Riester, Ralf Dressel, Daniel Langenstroth, Jörg Gromoll, Erika Sasaki, Rüdiger Behr

**Affiliations:** 1 Stem Cell Biology Unit, German Primate Center—Leibniz Institute for Primate Research, Göttingen, Germany; 2 Microarray and Deep-Sequencing Core Facility, University Medical Center Göttingen (UMG), Göttingen, Germany; 3 Department of Cellular and Molecular Immunology, University of Göttingen, Göttingen, Germany; 4 Centre of Reproductive Medicine and Andrology, University of Münster, Münster, Germany; 5 Department of Applied Developmental Biology, Central Institute for Experimental Animals, Kawasaki-ku, Kawasaki, Kanagawa, Japan, Keio Advanced Research Center, Keio University, Shinjuku-ku, Tokyo, Japan; 6 DZHK (German Center for Cardiovascular Research), Partner Site Göttingen, Göttingen, Germany; University of Newcastle upon Tyne, UNITED KINGDOM

## Abstract

Groundbreaking studies showed that differentiated somatic cells of mouse and human origin could be reverted to a stable pluripotent state by the ectopic expression of only four proteins. The resulting pluripotent cells, called induced pluripotent stem (iPS) cells, could be an alternative to embryonic stem cells, which are under continuous ethical debate. Hence, iPS cell-derived functional cells such as neurons may become the key for an effective treatment of currently incurable degenerative diseases. However, besides the requirement of efficacy testing of the therapy also its long-term safety needs to be carefully evaluated in settings mirroring the clinical situation in an optimal way. In this context, we chose the long-lived common marmoset monkey (*Callithrix jacchus*) as a non-human primate species to generate iPS cells. The marmoset monkey is frequently used in biomedical research and is gaining more and more preclinical relevance due to the increasing number of disease models. Here, we describe, to our knowledge, the first-time generation of marmoset monkey iPS cells from postnatal skin fibroblasts by non-viral means. We used the transposon-based, fully reversible *piggyback* system. We cloned the marmoset monkey reprogramming factors and established robust and reproducible reprogramming protocols with a six-factor-in-one-construct approach. We generated six individual iPS cell lines and characterized them in comparison with marmoset monkey embryonic stem cells. The generated iPS cells are morphologically indistinguishable from marmoset ES cells. The iPS cells are fully reprogrammed as demonstrated by differentiation assays, pluripotency marker expression and transcriptome analysis. They are stable for numerous passages (more than 80) and exhibit euploidy. In summary, we have established efficient non-viral reprogramming protocols for the derivation of stable marmoset monkey iPS cells, which can be used to develop and test cell replacement therapies in preclinical settings.

## Introduction

Takahashi and Yamanaka established in groundbreaking studies transcription factor-mediated reprogramming of fibroblasts to pluripotency [1,2]. This was achieved by the ectopic expression of just four transcription factors, namely OCT4, SOX2, KLF4 and c-MYC (OSKM). The open reading frames encoding OSKM were delivered to the cells by retroviral vectors, which stably and irreversibly integrate into the genome of the cells. Retrovirus-mediated expression is very robust and stable, yet there are two major disadvantages: firstly, the integrated proviruses may induce harmful mutations. Secondly, the activity of the viral promoters used to drive transgene expression could lead to aberrant permanent activation of endogenous genes in the genomic vicinity of the insertion site in the reprogrammed cells. This may also include activation of oncogenes resulting in an increased tumorigenic potential of the induced pluripotent stem (iPS) cells. Hence, although viral reprogramming is very robust and useful for experimental *in vitro* studies, insertion mutagenesis and the increased oncogenic potential are major drawbacks of this initial reprogramming approach with regard to the envisaged clinical application of iPS cells. Therefore, in the light of the potential use of iPS cells in cell replacement therapy, original retrovirus-based reprogramming is insufficient. Accordingly, alternative strategies to deliver the reprogramming factors were developed which either circumvent genomic insertion of the expression constructs or make use of reversibly integrating constructs. Approaches circumventing the integration of a vector include episomal plasmids [3–6], non-integrating viral vectors [7–11] or transfection of modified mRNA encoding the reprogramming factors [12–16]. Also protein-, miRNA- and small molecule-driven reprogramming were published [17–21]. Although generally very promising, at least some of the latter approaches are currently rather experimental and do not work robustly. The excisable vectors included loxP site-flanked retroviruses. However, even after excision, a loxP site remains in the genome, still causing a mutation. Kaji et al. [22] and Woltjen et al. [23] published a very attractive alternative approach: *piggyBac* transposon-mediated reprogramming. The suitability of the *piggyBac* system for reprogramming was demonstrated in human and mouse fibroblasts. This system has several great advantages compared to other reprogramming strategies: (i) it can stably integrate into the host cell genome and is replicated during cell division, resulting in stable expression of the reprogramming factors even over long periods of time and over many generations of cells, (ii) it can deliver large DNA fragments up to 12 kb, and (iii) most importantly, it is fully reversible, i.e. it can be excised from the host cell genome without leaving any footprint in the genome after excision. Hence, iPS cells with a completely unmodified genome compared to the initially reprogrammed cells, except epigenetic changes, can be obtained. This is in contrast to other types of excisable / mobile transposons [24,25]. In fact, to our knowledge the *piggyBac* transposon is the only mobile genetic element combining stable integration with complete reversibility.

Cell replacement therapies based on pluripotent stem cells may become an important option for the treatment of currently incurable diseases associated with the degeneration of a specific cell type or a tissue [26–28]. However, these novel approaches must be carefully tested since the use of pluripotent stem cells during cell replacement therapies is associated with the risk of teratoma formation. Therefore, besides the requirement of efficacy testing also the safety of the therapy needs to be carefully evaluated. In this context, it is important to test cell replacement therapies preclinically in a model species that closely resembles human physiology, immunology, genetics and anatomy and further allows testing over long periods of time. For this reason, we chose the common marmoset monkey (*Callithrix jacchus*) as a non-human primate species to generate iPS cells. The common marmoset is frequently used in biomedical research and recently gained increasing interest especially in the fields of stem cell research [29,30], reproductive biology [31–33] and neurobiology (reviewed in [34,35]). Its small size—yet representing primate physiology-, the absence of zoonoses, easy handling and relatively low housing costs are some of its important practical advantages further promoting the marmoset monkey as a bridging non-human primate species between rodent studies and clinical application. However, previously, it was shown that the four classical reprogramming factors KLF4, c-MYC, SOX2 and OCT4 are not sufficient for the derivation of stably reprogrammed marmoset iPS cells even when lentiviral vectors were used. Instead, two additional pluripotency factors were employed to stably reprogram marmoset monkey cells: the transcription factor NANOG and the RNA binding protein LIN28 [36]. Here, we describe, to our knowledge, the first-time generation of marmoset monkey iPS cells from postnatal skin fibroblasts using a six-factor-in-one-construct *piggyBac* system. We characterized six individual iPS cell lines in comparison with marmoset monkey embryonic stem cells.

## Material and Methods

### Animals and animal housing; ethics statement

Marmoset monkeys (*Callithrix jacchus*) for this study were obtained from the self-sustaining breeding colony of the German Primate Center (Deutsches Primatenzentrum; DPZ). The German Primate Center is registered and authorized by the local and regional veterinary governmental authorities (Reference number: 122910.3311900, PK Landkreis Göttingen). The legal guidelines for the use of animals and the institutional guidelines of the DPZ for the care and use of marmoset monkeys were strictly followed. Health and well-being of the animals of the colony were controlled daily by experienced veterinarians and animal care attendants. Marmoset monkeys are social tree-living New World monkeys originating from the tropical northeast of Brazil. Accordingly, the animals were pair-housed in a temperature- (25 ± 1°C) and humidity-controlled (65 ± 5%) facility. These parameters were controlled daily. Room air was changed several times per hour and filtered adequately. Illumination was provided by daylight and additional artificial lighting on a 12.00:12.00 hour light:dark cycle. Each cage consisting of stainless steel had a vertical orientation [180 cm (height) × 65 cm (width) × 80 cm (depth)] and was furnished with wooden branches and shelves for environmental enrichment and contained a wooden sleeping box mimicking the monkeys’ natural habitats best. The housing room and the cages were cleaned with water and disinfectant at weekly intervals. The animals were fed ad libitum with a pelleted marmoset diet (ssniff Spezialdiäten, Soest, Germany). In addition, 20 g mash per animal was served in the morning and 30 g cleanly cut fruits or vegetables mixed with noodles or rice were supplied in the afternoon. Furthermore, once per week mealworms or locusts were served in order to provide adequate nutrition. Drinking water was always available. All materials are changed regularly, cleaned and sterilized. Marmoset monkey fibroblasts were obtained from two neonatal animals. The ages and the body weights of the animals (one male: animal #16008, one female: animal #16384) were one day post-partum and ~33 g, respectively. In captivity, marmosets often give birth to triplets or even quadruplets. However, the mother is usually able to feed and rear only two neonates, which is the normal litter size of free-living marmosets. Therefore, the neonates from triplet births were used to collect skin for this study and additional organs for other scientific purposes. Neonatal marmosets were selected based on the body weight. The ones that had lowest birth weight were selected for euthanasia. All animals were narcotized with Pentobarbital (Narcoren; 0.05 ml intramuscular) by a veterinarian and euthanized with an intracardial injection of 0.5 ml Pentobarbital before a lack of nourishment caused evident suffering of the animals.

Animal experiments to obtain different developmental stages of marmoset monkeys were approved by an external ethics committee (Niedersächsisches Landesamt für Verbraucherschutz und Lebensmittelsicherheit, AZ 42502–04–12/0708). For this study, small pieces of skin were used from euthanized neonatal marmosets. Wherever applicable, the ARRIVE guidelines were followed.

### Cell culture

#### Isolation of common marmoset primary fibroblasts

Pieces of skin were washed with PBS and shaved. The skin was cut into pieces of 1x1 mm—2x2 mm and incubated in DMEM/5 mg/mL Collagenase type IV (Gibco) for 1 to 3h at 37°C in a Thermomixer (Eppendorf) at 800 rpm. After complete tissue dissociation, cells were pelleted (5 min, 300 × g, RT). The supernatant was aspirated, and the cells were seeded in fresh M10 medium [DMEM (Gibco), 10% (v/v) Fetal Bovine Serum (Gibco), 1% (v/v) Penicilline/Streptomycine (Gibco), 0,25 μg/mL Amphotericin B (Sigma), 1% (v/v) MEM Non-Essential Amino Acids Solution (Gibco), 2 mM GlutaMAX (Gibco)] and cultured at 37°C and 5% CO_2_. TrypLE Express (Gibco) was used for passaging.

#### Mouse embryonic fibroblasts (MEFs)

Gamma-irradiated MEFs served as feeder cells. E12.5 embryos from CD1 mice were washed in Ca^2+^/Mg^2+^-free PBS (PAA). Head and hematopoietic organs, during that stage mainly the liver, were removed; remaining tissue was minced into small pieces and washed in PBS and then incubated in 0,05% Trypsin-EDTA at 37°C for 20 min. Per 25 mL reaction, 5 mL FBS, 20 mL M10 and DNaseI (Roche, 100 Kunitz units/mL), were added. After 10 min incubation at 37°C, cells were pelleted (15 min, 300 × g, RT) and seeded in fresh M10 medium. Prior to cryopreservation in 90% FBS/10% DMSO, cells (passage 0 to 4) were irradiated with 30 Gy (5 Gy/min). MEFs were thawed and seeded in M10 medium on gelatin coated cell culture dishes one or two days prior to use at a density of 50,000 to 100,000 cells per cm^2^. MEFs provided a stable feeder cell layer for 30–80 days.

#### Nucleofection

Primary fibroblasts from newborn common marmoset monkeys were transfected using the Nucleofector 2b Device (Lonza) with the Amaxa Basic Nucleofector Kit for Primary Mammalian Fibroblasts and program V-013 or using the 4D Nucleofector System with buffer 2 and program CA-137.

#### Reprogramming procedure

After nucleofection, fibroblasts were cultured in M10 medium for three days or, depending on the transfected reprogramming construct, in M10 medium with 1 μg/mL puromycin for five days on standard cell culture dishes, then transferred onto MEFs (2,0 × 10^5^ (unselected) or 0,2 × 10^5^ (selected) cells per Ø10cm culture dish) and cultured in embryonic stem cell medium (ESM) [KO-DMEM (Gibco), 20% (v/v) KnockOut Serum Replacement (Gibco), 1% (v/v) Penicilline/Streptomycine (Gibco), 0,25 μg/mL Amphotericin B (Sigma), 1% (v/v) MEM Non-Essential Amino Acids Solution (Gibco), 2 mM GlutaMAX (Gibco), 50 μM 2-mercaptoethanol (Gibco)] under hypoxic conditions (37°C, 8% CO_2_, 5% O_2_). During the first six days of culture on MEFs, ESM was supplemented with 2 mM valproic acid (Calbiochem). From day 30 on, individual colonies were picked manually. For further passaging of the putative iPS cells, StemPro Accutase (Life Technologies) was used.

### Cloning / Plasmid construction

Open reading frames of the reprogramming factors were PCR-amplified (KOD Hot Start DNA Polymerase, Novagen) from P51 single strand cDNA of the common marmoset ESC line cjes001 [37] and cloned into the plasmid vector pBluescriptIISK- (Stratagene). Oligonucleotides (Sigma) introducing restriction sites and the respective restriction enzymes (New England Biolabs) are listed in Table 1.

**Table 1:** Oligonucleotides used for the cloning of the reprogramming factors.

Gene	Primer name (orientation), primer sequences (5ˈ → 3ˈ)	Restriction enzymes
***SOX2***	G0028 (fwd): GCTAGGATCCACAGCGCCCGCATG	BamHI/XhoI
	G0030 (rev): CCGCTCGAGAATGCCTCCCCCGTCCAGTTCG	
***OCT4***	G0022 (fwd): GATCGGATCCTTGGGGCGCCTTCCTTC	BamHI/XbaI
	G0023 (rev): CTGATCTAGACTCCTCTCCCTGTCCCCC	
***KLF4***	G0020 (fwd): GTACGGATCCTGCGCAGCCACCTGGC	BamHI/XbaI
	G0021 (rev): GTACTCTAGACAGTGTGGGTCATATCCACTG	
***c-MYC***	G0077 (fwd): ATAAGAATGCGGCCGCAATGCCCCTCAACGTCAGCTTC	NotI/SalI
	G0078 (rev): ATGGCCGACGTCGACTTATGCACAAGAGTTCCGCAGC	
***LIN28***	G0024 (fwd): GATCGGATCCGGCCACGGGCTCAGCCG	BamHI/XhoI
	G0025 (rev): GACTCTCGAGATAGCCAAAGAATAGCCCC	
***NANOG***	G0018 (fwd): GATCAAGCTTCCTTTTCCCCAATAATAACATG	HindIII/XhoI
	G0019 (rev): CTGACTCGAGTAGTGTCAGTTTCATTCATC	

**Fig 1:**
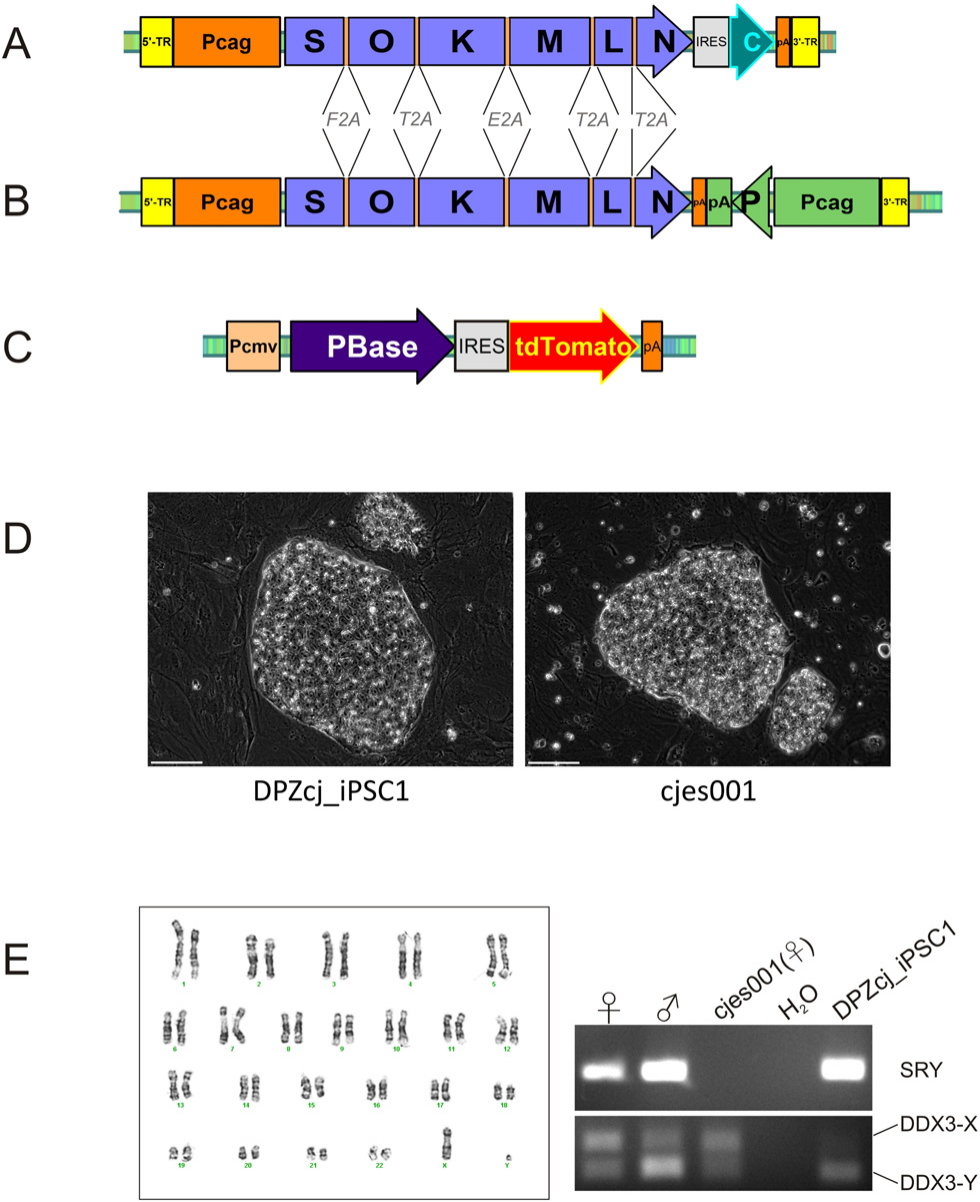
Reprogramming constructs and morphology of marmoset monkey iPS cells. **A) and B**) Constructs containing the reprogramming cassette. Expression of the reprogramming factors is driven by the CAG promoter (Pcag). Stop codons of the first five reprogramming factors were substituted with coding sequences for 2A peptides (F2A, T2A, E2A). IRES, internal ribosomal entry site; pA, poly A signal; 5ˈ-TR, 5ˈ-terminal repeat; 3ˈ-TR, 3ˈ-terminal repeat; S, *SOX2*; O, *OCT4*; K, *KLF4*; M, *c-MYC*; L, *LIN28*; N, *NANOG*; C, Cerulean; P, puromycin resistance gene. **C)** Expression of the Transposase (PBase) is driven by the Cytomegalovirus promoter (Pcmv). **D)** Morphology of iPS cell colonies. The morphology of the generated iPSCs (DPZcj_iPSC1) and the marmoset ES cell line cjes001 are indistinguishable from each other. Bars = 100 μm **E)** Karyotype analysis of DPZcj_iPSC1. Karyogram showing a normal Karyotype 46, XY (left). Right: PCR analysis confirming the male genotype. Primers used amplify a fragment of the Sex-determining region Y gene (*SRY*) and the X or Y chromosome-linked genes *DDX3* (*DDX3-X*, *DDX3-Y*). The analysis was done with genomic DNA from a newborn female marmoset (♀), a newborn male marmoset (♂), an established female ES cell line (cjes001) and the generated iPS cell line DPZcj_iPSC1. Water was used as negative control (H_2_O).

Single elements were combined into the reprogramming constructs pTT-PB-SOKMLN-Cer or pTT-PB-SOKMLNpuro using the plasmid vector pTRE-Tight (Clontech) as backbone. The stop codons in the ORFs of *SOX2* (S), *OCT4* (O), *KLF4* (K), *c-MYC* (M) and *LIN28* (L) were deleted and the ORFs were joined with coding sequences for 2A peptides. After the stop codon for *NANOG* (N), either an IRES and the ORF for Cerulean (pTT-PB-SOKMLN-Cer) or a puromycin resistance gene under control of a separate CAG promoter in reverse orientation (pTT-PB-SOKMLNpuro) were inserted. Expression of the multi-factor cassette is under control of the CAG promoter [38,39] (see also Fig. 1A, B and C).

For transposition of the reprogramming cassette, the ORF for a hyperactive, codon-optimized PBase [40] followed by an IRES and the ORF for the fluorescent protein tdTomato were cloned into the MCS of the expression vector pcDNA3.1(+) (Invitrogen).

Sequences of the reprogramming constructs were deposited in GenBank (http://www.ncbi.nlm.nih.gov/genbank/) and are available under accession numbers KM279352 (pTT-PB-SOKMLN-Cer) and KM279353 (pTT-PB-SOKMLNpuro).

### PCR for the detection of the reprogramming cassette and endogenous marker genes

Oligonucleotides (Sigma) used for detection of mRNA coding for endogenous and exogenous factors are listed in Table 2. KOD Hot Start DNA Polymerase from Merck was used.

**Table 2:** Oligonucleotides used for the detection and discrimination of exogenous and endogenous sequences and oligonucleotides used for real-time qPCR.

Fragment	Primer name (orientation), primer sequences (5ˈ → 3ˈ)	PCR product (bp)
Endogenous factors		
***SOX2***	G0086 (fwd): TCTTCCTCGCACTCCAGGGC	228
	G0030 (rev): CCGCTCGAGAATGCCTCCCCCGTCCAGTTCG	
***OCT4***	G0022 (fwd): GATCGGATCCTTGGGGCGCCTTCCTTC	510
	G0035 (rev): CAGGGTGATCCTCTTCTGCTTC	
***KLF4***	G0091 (fwd): GGAAGACGATCTTGGCCCCG	323
	G0021 (rev): GTACTCTAGACAGTGTGGGTCATATCCACTG	
***c-MYC***	G0079 (fwd): ATAAGAATGCGGCCGCACTGGATTTTTTTCGGGCAGTGG	456
	G00142 (rev): CCTGGATGATGATGTTTTTGATG	
***LIN28***	G0305 (fwd): GACGAGCTGTACAAGGGGAGTGAGAGGCGGCCAAAGGGG	334
	G0025 (rev): GACTCTCGAGATAGCCAAAGAATAGCCCC	
***NANOG***	G0018 (fwd): GATCAAGCTTCCTTTTCCCCAATAATAACATG	754
	G0075 (rev): TTATAGAAGGGACTGCTCCAGG	
Reprogramming cassette		
***SO (SOX2-OCT4)***	G0086 (fwd): TCTTCCTCGCACTCCAGGGC	735
	G0035 (rev): CAGGGTGATCCTCTTCTGCTTC	
***IC (IRES-Cerulean)***	G0095 (fwd): AGCGACCCTTTGCAGGCAGC	1073
	G0039 (rev): GAAGATCTACTTGTACAGCTCGTCCATG	
***PCAG-SOX2***	G0244 (fwd): GGGGACGGCTGCCTTCGG	190
	G0378 (rev): GCTCGGTACCAAGCTTAAG	
***PCAG-SOX2***	G0244 (fwd): GGGGACGGCTGCCTTCGG	361
	G0395 (rev): CGGTCGGGGCTGTTCTTCTG	
***NANOG-IRES***	G0069 (fwd): GGTTCCAGAACCAGAGAATGAAATC	576
	G0098 (rev): CACCGGCCTTATTCCAAGCG	
***Cerulean***	G0618 (fwd): ACGTAAACGGCCACAAGTTCAGC	211
	G0619 (rev): CCTTCGGGCATGGCGGACTTG	
Control fragments		
***mmSox2***	G0656 (fwd): GGACATGATCAGCATGTACCTCC	221
	G0657 (rev): TCTCCTCTTTTTGCACCCCTCC	
***β-ACTIN***	G0336 (fwd): GACGACATGGAGAAGATCTGG	562
	G0337 (rev): GGAAAGAAGGCTGGAAGAGTG	
Real-time qPCR		
***18S rRNA***	G0871 (fwd): ATTAAGGGTGTGGGCCGAAG	81
	G0872 (rev): GAGTTCTCCTGCCCTCTTGG	
***OCT4_endo (isoform A and B)***	G0963 (fwd): GCCAGGGCTTTTAGGATTAAGTT	68
	G0964 (rev): TGCCCTCACCCTTTGTGTTC	
***OCT4A_endo+exo***	G0877 (fwd): CCCCTGGTGCCGTGAAG	82
	G0878 (rev): TTCTGCAGAGCTTTGATGTCTTG	
***NANOG***	G0965 (fwd): ATGCCACCTGAAGATGTGTGAA	69
	G0966 (rev): TCAGCCAGTGCTCAGAGTGAA	
***Transposon***	G0961 (fwd): AGCCAACATACTTTCGGGAGGA	135
	G0962 (rev): TACTCATTGGGCCAGGATTCTC	
***SOX2***	G0969 (fwd): TTGTTCAAAAAAGTATCAGGAGTTGTC	97
	G0970 (rev): CTCTCCGTCCCCGTCTTAAAG	
***LIN28***	G0682 (fwd): GCACAGGGAAAGCCAACATAC	126
	G0683 (rev): CGAAACTTCCTGATAGCCAAAGA	

### Real-time quantitative PCR

Total RNA was extracted using the RNeasy Mini Kit (Qiagen, Hilden, Germany) following the manufacturer’s instructions. The genomic DNA was removed by using RNase-Free DNase (Qiagen). For cDNA synthesis, 1 μg of the isolated total RNA was reverse transcribed with Oligo(dT)18 primers using the Omniscript RT Kit 200 (Qiagen) following the manufacturer´s instructions. Real-time qPCR was performed on a StepOnePlus System (Applied Biosystems, Carlsbad, USA). For each PCR, cDNA transcribed from 10 ng mRNA served as template using Power SYBR Green PCR master mix (Applied Biosystems) and specific primers in a concentration of 600 nM (for primer sequences see Table 1). Each qPCR amplification was measured in triplicates, data were normalized against the housekeeping gene 18S rRNA. All data are presented as means±SEM.

### Embryoid body (EB) formation

After colonies were detached from the feeder cell layer with 1 mg/mL Collagenase type IV (dissolved in KO-DMEM, Gibco), they were pelleted (200 × g, 5 min, RT), gently re-suspended in ESM and incubated on a standard cell culture dish for 1h to allow residual feeder cells to attach. The cell suspension was then transferred to a non-adherent petri dish to allow cell aggregation in suspension. The medium was changed every other day carefully preventing removal of aggregating cells. After five to six days, the cell suspension was transferred to a gelatin or fibronectin coated cell culture dish to allow EBs to attach. Until fixation of the EBs (after another eight days in culture), medium was changed every other day. Incubation was always at 37°C and 5% CO_2_.

### Immunofluorescence

Cells were washed twice with PBS, fixed in PBS/2% (w/v) PFA/0,02% (v/v) TritonX-100 (30 min, RT) and washed twice with PBS/5% (w/v) BSA. Incubation with the first antibody diluted in PBS/5% (w/v) BSA was done overnight at 4°C. Cells were washed twice with PBS/5% (w/v) BSA and Alexa488 or Alexa594-coupled secondary donkey antibodies (Life Technologies) diluted 1:200 in PBS/5% (w/v) BSA were applied for 30 min at RT. Cells were washed twice with PBS/DAPI and coated with Citifluor mountant medium (CITIFLUOR ltd.). Images were taken with a Zeiss Observer Z1 (Zeiss). Antibodies and their dilutions were SSEA-4 (Millipore/Chemicon, MAB4304, 1:50), TRA-1–60 (eBioscience, 14–8863, 1:50), TRA-1–81 (eBioscience, 14–8883, 1:50), SALL4 (abcam, ab57577, 1:200), SOX2 (Millipore, AB5603, 1:50 and Cell Signaling, 3728, 1:200), OCT4 (Santa cruz, sc-8628, 1:50 and Cell Signaling, 2890, 1:100), KLF4 (R&D, AF3640, 1:50), NANOG (Cell Signaling, 4893, 1:100 and Cell Signaling, 4903, 1:300), LIN28A (Cell Signaling, 3978S, 1:70), CHD1 (BETHYL, A301–218A, 1:200), UTF1 (Millipore, MAB4337, 1:1000), β-Tubulin III (Sigma, T8660, 1:400), AFP (Dako, A0008, 1:200), SMA (Sigma, A2547, 1:750).

### Teratoma formation and histological analysis

For teratoma formation, 4.8 × 10^5^ cells from the cell line DPZcj_iPSC1 (P16) were injected subcutaneously into the inguinal region of male immunodeficient RAG2^-/-^γc^-/-^mice. Teratoma was retrieved 16 weeks after injection. Antibodies for immunohistological stainings of the teratoma were against β-Tubulin III (Sigma, T8660, 1:600) as ectodermal marker, SMA (Sigma, A2547, 1:1000) as mesodermal marker and SOX9 (Millipore, AB5535, 1:1000) as protein expressed in endodermal epithelium. Immunohistochemical staining procedure was as described previously [41].

### Transcriptome analysis

Transcriptome analysis, including RNA preparation, was done by the Transkriptomanalyselabor, Microarray and Deep-Sequencing Facility, University Medical Center Göttingen. RNA was isolated using the TRIzol Reagent (Life Technologies) according to manufacturer instructions. RNA quality was assessed by measuring the RIN (RNA Integrity Number) using an Agilent 2100 Bioanalyzer (Agilent Technologies, Palo Alto, CA). Library preparation for RNA-Seq was performed using the TruSeq RNA Sample Preparation Kit (Illumina, Cat. N°RS-122–2002) starting from 500 ng of total RNA. Accurate quantitation of cDNA libraries was performed by using the QuantiFluor dsDNA System (Promega). The size range of final cDNA libraries was determined applying the DNA 1000 chip on the Bioanalyzer 2100 from Agilent (280 bp). cDNA libraries were amplified and sequenced by using the cBot and HiSeq2000 from Illumina (SR; 1x50 bp; 5–6 GB ca. 30–35 million reads per sample). Sequence images were transformed with Illumina software BaseCaller to bcl files, which were demultiplexed to fastq files with CASAVA v1.8.2. Quality check was done via fastqc (v. 0.10.0, Babraham Bioinformatics). The Alignment was performed using Bowtie2 v2.1.0 to the cDNA for *Callithrix jacchus*. Data were conversed and sorted by samtools 0.1.19 and reads per gene were counted via htseq version 0.5.4.p3. Data analysis was performed using R/Bioconductor (3.0.2/2.12) loading DESeq, gplots and goseq packages. Candidate genes were filtered to a minimum of 4x fold change and FDR-corrected pvalue<0.05. For functional analysis gene ontology enrichment was tested accounting for gene length via R-package goseq. The data discussed in this paper are generated in compliance with the MIAME guidelines and have been deposited in NCBI’s Gene Expression Omnibus and are accessible through GEO Series accession number GSE64966.

### Karyotyping

Karyotyping was performed by the Cytogenetic Laboratory in the Department of Human Genetics at the Universitätsklinikum Hamburg-Eppendorf (Germany) according to standard procedures.

### Promoter methylation assay

Genomic DNA from cultured cells of the DPZcj_iPSC line 1, the ES cell line cjes001 and of marmoset fibroblasts (from three different passages each) was isolated and bisulfite converted with the Epitect Bisulfite Kit (Qiagen) according to provided protocols. To control if the DNA bisulfite conversion was complete, control dispensations at non CpG sites were performed during subsequent pyrosequencing assays. Specific sequences from promotors of *OCT4* (chromosome 3; 31633134–31632640, NCBI version 13-Feb-2013), the germ cell marker genes *VASA* (chromosome 2; 151940913–151940610, NCBI version 17-Feb-2013) and *MAGE A-4* (chromosome X; 138852177–138852680, NCBI version 13-Feb-2013) and the imprinted genes *H19* (chromosome 11; 128443800–128444200, NCBI version 9-Dec-2012) and *MEST* (chromosome 8; 115762911–115762510, NCBI version 17-Feb-2013) were subsequently amplified using the PyroMark PCR Kit (Qiagen). PCRs were performed in volumes of 20 μl with 10 μl provided master mix, 1 μl (20 pmol) of forward and reverse primers and 3 μl bisulfite converted DNA template applying the following program: One cycle of 95°C for 15 min, 45 cycles of 95°C for 15 s, 56°C for 20 s and 72°C for 30 s and one cycle of 72°C for 5 min. Forward and reverse primers which were used for PyroMark PCRs are listed in Table 3. Quantification of DNA methylation levels at differentially methylated CpG sites of amplified bisulfite converted promotor regions was performed by pyrosequencing using the PyroMark Q24 System (Qiagen). The sequencing primers are listed in Table 3. Analysis of pyrosequencing data and quantification of DNA methylation was performed with the PyroMark Q24 software (Qiagen). The accuracy and reproducibility of the assays was confirmed by analyzing control samples (sperm and blood) which exhibited specific expected levels of DNA methylation. Mean methylation levels of sperm DNA (n = 7) were high for *H19* (98%) and low for *MEST* (8%), *VASA* (6%), *MAGE A-4* (7%) and DNA methylation levels of blood *DNA* (n = 3–7) were intermediate for *H19* (64%) and *MEST* (62%) and high for *VASA* (98%), *MAGE A-4* (90%) and *OCT4* (90%).

**Table 3:** Oligonucleotides used for DNA-methylation analyses.

Gene	Primer sequences (5ˈ → 3ˈ)	PCR product (bp)
***VASA***	Fwd: GTTGGAGTTAGTTATTAGTTATTTGTTGT	117
	Rev: AACTTACTCTCCCCAATCCC	
	Seq: AGTTATTTGTTGTTGGAG	
***H19***	Fwd: TGATTTGAGTATGGGGGGTGGATTAG	168
	Rev: CTATTCCCAAATAACCCCCATAA	
	Seq: AGTTTATTTTAGTTGGGTTT	
***MAGE A-4***	Fwd: TGATTTGAGTATGGGGGGTGGATTAG	204
	Rev: ACCCACCAATAACCCAAAACAACCAACA	
	Seq: GGGGGGTGGATTAGA	
***MEST***	Fwd: GGAAAGAGGGGGTGTGGTTG	155
	Rev: ACAAAAATAACATCCCCTTCTCA	
	Seq: TGGTAGTTTAGGGATTAGGGT	
***POU5F1***	Fwd: GGAGAGAGGGGTTGAGTAGTTT	181
	Rev: ACCAAATCCCAAAATCAACCCAACCTAT	
	Seq: GGTAAGTTTTTATTTTATTAGGTTT	

## Results

### Non-viral reprogramming by the *piggyBac* transposon/transposase system

To generate virus-free marmoset monkey iPS cells, we made use of the *piggyBac* transposon/transposase [42] system which was shown to be suitable for the generation of human and mouse iPS cells as a four-factor-construct [22,23]. Since it was shown that the four classical reprogramming factors *KLF4*, c-*MYC*, *SOX2* and *OCT4* are not sufficient for the derivation of stably reprogrammed marmoset iPS cells even when lentiviral vectors were used, we cloned all six marmoset monkey factors and constructed completely novel expression constructs containing all six factors in one cassette (Fig. 1A, B) plus a selection marker. In order to ensure that all factors are expressed in each individual cell and to reduce the number of integration sites (compared to an approach using single factor vectors), the stop codons of *SOX2*, *OCT4*, *KLF4*, *c-MYC* and *LIN28* were replaced by coding sequences for 2A peptides. These peptides induce co-translational separation of individual proteins encoded by one mRNA [43–45]. The stop codon of *NANOG* is followed by an IRES and the open reading frame (ORF) for the fluorescent protein Cerulean (Fig. 1A). Expression of the whole cassette is driven by the CAG promoter [38,39], and the cassette is flanked by *piggyBac* inverted repeats. Selection of primary reprogrammed cell colonies relying on Cerulean fluorescence, however, was insufficient because the IRES-mediated expression of the fluorescent protein seemed not to be robust. In order to obtain a more effective selection system, we decided to replace Cerulean as selection marker by a puromycin resistance cassette (Fig. 1B). To achieve this, the puromycin resistance gene was cloned in an inverted orientation into the original expression cassette and put under the control of a separate CAG promoter (Fig. 1B). Both ORFs were followed by polyadenylation signals. Hence, the modified construct shown in Fig. 1B contains two independent genes. To ensure maximal integration efficiency, a codon optimized hyperactive PBase was used [40]. Expression of PBase is driven by the CMV promoter and the PBase ORF is linked to an IRES followed by the ORF for the fluorescent protein tdTomato (Fig. 1C). Using the first expression construct (Fig. 1A), we generated one iPS cell line (DPZcj_iPSC1) with morphology indistinguishable from the morphology of the established marmoset ES cell line cjes001 [37] (Fig. 1D). Five additional iPS cell lines (DPZcj_iPSC2–6) were derived after puromycin selection of nucleofected fibroblasts. All cell lines exhibited the typical ES cell colony morphology when grown on mouse embryonic fibroblasts as feeder cells (S1 Fig.). As representative of the six iPS cell lines we characterized the line DPZcj_iPSC1 in detail. The other five cell lines were characterized by selected assays.

### Culture of iPS cells

In our established system, first primary colonies appear approximately 16 days after transfected fibroblasts were transferred onto feeder cells. Macroscopically visible colonies with a morphology resembling common marmoset embryonic stem cells can be picked from ~day 30 onwards. The time point for the appearance of the colonies seems to be solely dependent on the duration of culture on feeder cells. The duration of feeder-free culture after nucleofection before cells were transferred onto MEFs varied in different experiments and had no influence on the period necessary for colony formation on feeders. However, supplementation of the media with valproic acid in the initial phase of reprogramming seems to be strongly supportive of colony formation.

All six lines generated have been in culture for more than 11 months, the first line even for more than 2 years and over 80 passages. All generated cell lines were cryopreserved and result in viable and well-proliferating cell cultures after thawing.

### The DPZcj_iPSC1 line is male

The iPS cell line DPZcj_iPSC1 still had a normal male karyotype (46, XY) after 68 passages in culture (Fig. 1E left panel). Previously described iPS cell lines were all female [36,46,47]. PCR analysis of the Y-chromosomal gene Sex-determining region of Y chromosome (*SRY*) confirmed that the iPS cell line DPZcj_iPSC1 characterized in this study is male (Fig. 1E right). Primers specific for *SRY* amplified short fragments of genomic DNA (gDNA) of both, phenotypically male and female common marmoset monkeys with a stronger signal intensity using male DNA. The “male-specific” signal in female tissues is due to a cross-sex-cell chimerism between siblings, which develops by placental anastomoses during early pregnancy and has been described earlier [48,49]. PCR on gDNA from the generated iPS cell line indicated a male genotype, while the established marmoset ES cell line cjes001, which was used as reference, was female [37] (Fig. 1E right panel).

### Induction of endogenous pluripotency markers

**Fig 2:**
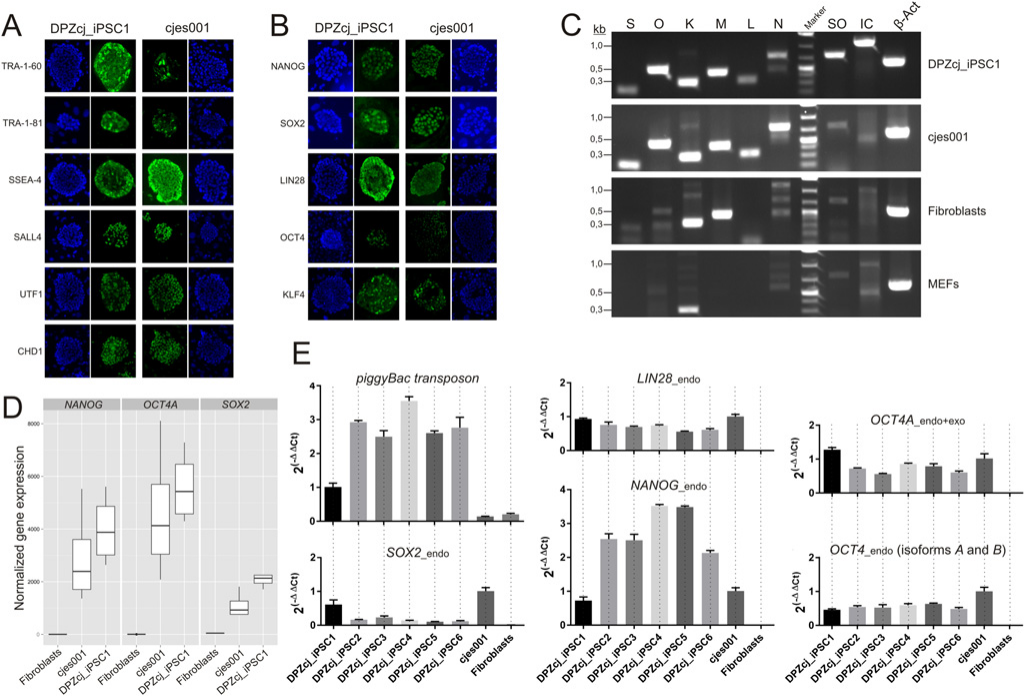
Expression of pluripotency markers. **A and B**) Immunofluorescence staining of iPS cell colonies. Immunofluorescence shows similar expression of several pluripotency factors in the generated iPSCs and in the ES cell line cjes001 indicating reactivation of the endogenous pluripotency factors in the iPS cell line (left). Also expression of the reprogramming factors delivered by the *piggyBac* transposon is detectable. **C)** PCR analysis of the generated iPSCs for expression of endogenous reprogramming factors. Primer pairs specific for endogenous factors (S, O, K, M, L, N) or the reprogramming cassette (SO, IC) were used. Analysis was done with cDNA from the generated iPS cell line DPZcj_iPSC1, an established ES cell line (cjes001), primary fibroblasts and mouse feeder cells (MEFs). As loading control, a fragment of *β-ACTIN* was amplified. S, *SOX2*; O, *OCT4*; K, *KLF4*; M, *c-MYC*; L, *LIN28*; N, *NANOG*. The six reprogramming factors are expressed endogenously by the iPS cell line. As expected, *KLF4* and *c-MYC* were also detected in cDNA from primary fibroblasts. Because of high sequence similarity, *Klf4* was also amplified from mouse feeder cell cDNA. Expression of the reprogramming cassette was only detected in the iPSCs. **D)** Boxplot of *NANOG*, *OCT4* and *SOX2* normalized gene expression for fibroblasts, ESCs, and iPSCs. Only the pluripotency associated isoform of *OCT4* (*OCT4A*) was selected for comparison, although the other transcript isoforms show similar expression profiles (data not shown). The upper whisker extends from the hinge to the highest value that is within 1.5 * IQR of the hinge, where IQR is the inter-quartile range, or distance between the first and third quartiles. The lower whisker extends from the hinge to the lowest value within 1.5 * IQR of the hinge. Boxplot generated by ggplot2 [51]. **E)** Real-time PCR for pluripotency markers expressed ectopically from the reprogramming cassette and endogenously. Levels of mRNA for the reprogramming cassette (*Transposon*), *SOX2*, *LIN28*, *NANOG*, and *OCT4* in all six generated iPS cell lines (DPZcj_iPSC1–6) and in marmoset skin fibroblasts were compared to the levels in the established embryonic stem cell line cjes001. qPCR was performed on RNA isolated from triplicate cultures. The relative level ± SEM is shown for each cell line. To determine RNA levels of genes expressed endogenously, where possible, primer pairs were used with one of the primers annealing to an untranslated region of the mRNA.

We tested the expression of several surface pluripotency markers and of pluripotency-associated transcription factors that were not present in the reprogramming cassette. Immunofluorescence staining demonstrated the presence of tumor rejection antigen (TRA)-1–60, TRA-1–81 and of stage specific embryonic antigen 4 (SSEA-4) on the iPS cells (Fig. 2A, upper panels). As transcription factors associated with pluripotency, we tested expression of Sal-like protein 4 (SALL4), undifferentiated embryonic cell transcription factor 1 (UTF1) and chromodomain helicase DNA binding protein 1 (CHD1). All three factors were detected (Fig. 2A, lower panels). Also expression of the factors used for reprogramming (SOX2, OCT4, KLF4, LIN28 and NANOG) could be demonstrated on the protein level (Fig. 2B). As positive control, we used the ES cell line cjes001. Staining intensities were similar between the iPS cells and the ES cells with the exceptions of TRA-1–60 and TRA-1–81, which gave stronger signals in iPS cells (Fig. 2A). Since antibody staining cannot discriminate between an endogenous and exogenous origin of the protein, we performed PCR with primers specific for the untranslated regions of mRNAs derived from the endogenous genes and with primers specific for the *piggyBac* cassette-derived transcript. This analysis proved that *SOX2*, *OCT4*, *KLF4*, *c-MYC*, *LIN28* and *NANOG* were expressed endogenously by the generated iPS cells (Fig. 2C). Although expression of the reprogramming construct was still detectable on the transcript level (Fig. 2), Cerulean fluorescence was not visible in the iPS cell colonies (not shown). For quantification, we compared the total expression levels of the core pluripotency factors *NANOG*, *OCT4A* and *SOX2* [50] in the iPS cells (DPZcj_iPSC1), the ES cell line cjes001 and marmoset skin fibroblasts based on deep sequencing data. All three genes were higher expressed in iPS cells than in ES cells, while no expression was detected in fibroblasts (Fig. 2D). To discriminate between endogenous expression and exogenous expression from the reprogramming cassette and to compare relative expression levels of the reprogramming cassette between the different iPS cell lines, we performed real-time quantitative PCR analyses. Compared to DPZcj_iPSC1, expression of the reprogramming cassette was 2.5 to 3.5-fold higher in the remaining five iPS cell lines (DPZcj_iPSC2–6). The latter ones were in much lower passages when the analyses were performed than the DPZcj_iPSC1 line. Expression levels of endogenous *SOX2* were reversed compared to the expression level of the reprogramming cassette. Compared to ES cells, expression of endogenous *SOX2* was about 50% in DPZcj_iPSC1. Endogenous *SOX2* expression levels of the remaining iPS cells were in the range of 10–23% of the reference ES cell line. The expression levels of endogenous *LIN28* in the iPS cells were comparable to ES cells (range 56%- 93%). Expression levels of endogenous *NANOG* of the iPS cell lines 2–6 exceeded the respective expression level of ES cells 2.5 to 3.5-fold, while theDPZcj_iPSC1 line exhibited 70% of the ES cell *NANOG* expression. Due to a short 5′ UTR of the *OCT4* mRNA, it was impossible to design a reliable qPCR primer which specifically detected endogenous *OCT4A*. Hence, our analyses were limited to either simultaneous detection of both endogenous *OCT4* isoforms, i.e. *OCT4A* and *OCT4B*, or to the detection of the total mRNA encoding the pluripotency-associated *OCT4A* isoform, irrespective of its endogenous or exogenous origin. The total *OCT4A* mRNA expression level in the iPS cell line DPZcj_iPSC1 was slightly higher than that in the ES cells, confirming the quantification results obtained by deep sequencing (Fig. 2D), whereas it was slightly reduced in the five remaining iPS cell lines compared to the ES cell line. However, all mRNA expression levels were generally in the same range in the pluripotent cell lines, while, importantly, all factors were undetectable in fibroblasts.

Searching the ENSEMBL database for *OCT4* transcripts revealed an additional *OCT4* transcript (ENSCJAT00000038869) which contains the pluripotency-associated *OCT4A-*specific exon 1 and encodes a truncated OCT4A form consisting of only 174 amino acids instead of 360 amino acids. This truncated form, which differs only in 2 nucleotides from the normal *OCT4A* transcript, has to our knowledge not been functionally characterized. It is not possible to discriminate between the two isoforms by conventional PCR analyses; only TaqMan Assays would allow discrimination between them. In case that this transcript was also present in our cells, it was co-amplified with the primers designed to detect *OCT4A*. Total endogenous *OCT4* (*OCT4A* plus *B)* levels were slightly lower in the iPS cell lines compared to the ES cell line. None of the pluripotency factors analyzed was expressed in marmoset fibroblasts.

### Differentiation assays demonstrate pluripotency

**Fig 3:**
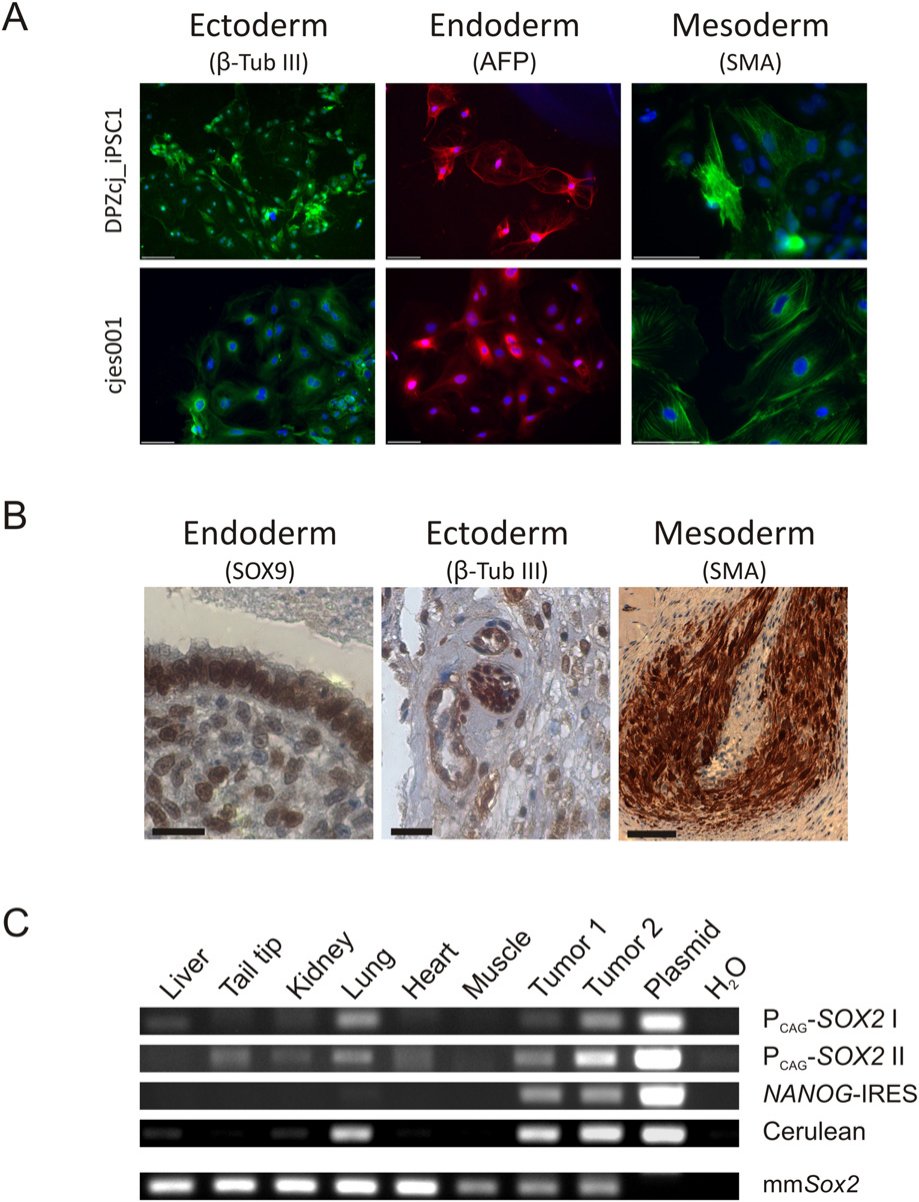
Testing of pluripotency. **A**) Immunofluorescence stainings of EB outgrowths. Embryoid bodies from the iPS cell line DPZcj_iPSC1 and an established ES cell line (cjes001) were plated and outgrowths were analyzed for expression of markers of the three germ layers: β-Tubulin 3 (β-Tub III), α-fetoprotein (AFP) and smooth muscle actin (SMA). EB outgrowths show positive staining for all markers. Antibodies were successfully tested also on native tissue by conventional immunohistochemistry (data not shown). Bars = 100 μm. **B)** Immunohistochemical analysis of a teratoma. Tumor tissue was analyzed for expression of markers of the three germ layers: SOX9, β-Tubulin 3 (β-Tub III), and smooth muscle actin (SMA). The tumor shows positive staining for all markers and was classified as teratoma. Bars = 100 μm **C)** PCR analysis of genomic DNA from the teratoma and several mouse tissues for presence of the reprogramming cassette. Two tissue pieces were dissected out of the teratoma (Tumor 1+2). As positive control, the reprogramming construct pTT-PB-SOKMLN (Plasmid) was used as template. Different primer pairs specific for the reprogramming cassette were used. As control, a fragment of the murine *Sox2* ORF (mm*Sox2*) was amplified. Injected cells seem to be present also at other sites than the injection site.

To investigate the *in vitro* differentiation potential of the iPS cells, embryoid bodies were generated, plated on cell culture dishes for 8 days, and differentiated outgrowths were analyzed for expression of markers of the three germ layers. The outgrowths showed immunofluorescence signals for the ectodermal marker β-Tubulin 3 (β-TUB III), the endodermal marker α-fetoprotein (AFP), as well as the mesodermal marker α-smooth muscle actin (SMA) (Fig. 3A). ES cells were used as positive control. To investigate the *in vivo* differentiation potential, we injected the iPS cells into RAG/C mice. 16 weeks after injection, one out of two mice developed a tumor at the injection site with a diameter of about 1 cm. The tumor tissue was histologically analyzed and expression of markers of the three embryonic germ layers was immunohistochemically analyzed. Detection of SOX9 (endodermal epithelium), β-Tubulin 3 (β-TUB III, ectoderm), and smooth muscle actin (SMA, mesoderm) indicates pluripotency (Fig. 3B).

### Injected iPS cells invade different tissues and organs apart the injection site

To confirm that the injected iPS cells developed the teratoma tissue, we performed PCR analysis of gDNA isolated from two tumor tissue samples taken from different sites of the tumor. We used multiple primer pairs specific for the reprogramming cassette. Besides teratoma tissue, we also analyzed liver, tail tip, kidney, lung, heart, and muscle of the recipient mouse (Fig. 3C). In both tumor biopsy samples (Tumor 1 and 2), the reprogramming cassette was detected with all four primer pairs tested. Importantly, the reprogramming cassette was also detected in mouse tissues remote from the injection site (inguinal region). The different PCR assays exhibited different sensitivities depending on the primer pairs. One of the primer pairs detected the reprogramming cassette in gDNA of all analyzed mouse tissues except for liver and muscle. Three out of four primer pairs detected the cassette in lung gDNA (Fig. 3C). The PCR analysis clearly shows that iPS cells (or at least genomic DNA from them) are present at other sites in the mouse body than the injection site. A preferred site of iPS cell settlement appears to be the lungs.

### The transcriptomes of the marmoset iPS cells and ES cells are similar

**Fig 4:**
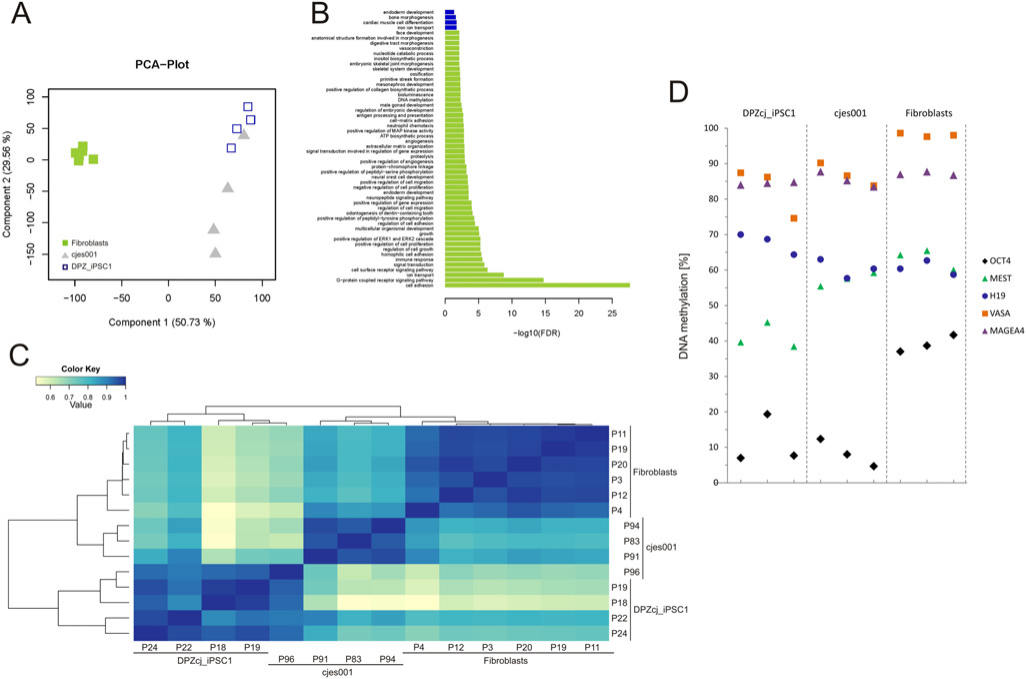
Functional transcriptomics and methylation analysis. **A)** Principle component analysis (PCA) plot. For a global view on the sample relationships a principal component analysis was computed based on one third of the genes with highest variance in expression levels. Principal component 1 and 2 account for 80% of the inherent data variance between the samples. Fibroblast and iPS cell samples are segregated from each other and cluster according to their origin. Solely ESC samples show higher data variance spread, but overlap with iPS cells. **B)** Gene ontology (GO) analysis. In order to test the functional association of the candidate genes, an enrichment test for GO terms was conducted for the comparisons fibroblasts-vs-DPZcj_iPSC1 (green bars) and cjes001-vs-DPZcj_iPSC1 (blue bars). For fibroblasts-vs-DPZcj_iPSC1 the top 50 GO terms with the best FDR-corrected p-values were chosen, for cjes001-vs-DPZcj_iPSC1 only four GO terms were found to be significant (pFDR<0.05). **C)** Heat map and hierarchical cluster of the normalized transcriptome expression profile for the iPS cell line DPZcj_iPSC1, the ES cell line cjes001 and marmoset skin fibroblasts. Darker color (blue) indicates correlation of gene expression. Fibroblasts and cjes001 cells exhibit the most similar gene expression, while iPS cells exhibit a distinct expression profile, further reflected in the cluster dendrogram. **D)** DNA promoter methylation analysis. In order to evaluate the epigenetic status of cells from the iPS cell line DPZcj_iPSC1, DNA methylation at specific CpG sites of the germ cell marker genes *VASA* and *MAGE A-4*, the imprinted genes *H19* and *MEST* and the pluripotency gene *OCT4* was determined and compared to cjes001 ES cells and fibroblasts. Data points represent DNA methylation levels for cells from one passage of the respective cell types.

Reprogramming of fibroblasts to iPS cells involves a massive change of the cellular program, which is reflected by the cells’ transcriptomes. To reveal global differences and similarities between primary fibroblasts initially used for reprogramming, established marmoset ES cells and the iPS cell line, whole transcriptome analyses were performed by deep sequencing. Principle component analysis (PCA) provides a general overview (Fig. 4A): Primary fibroblast samples closely clustered together. ES cells as pluripotency reference were clearly separated from the fibroblasts, although the ES cell transcriptomes had a higher data variance regarding component 2 than the transcriptomes of fibroblasts. Induced PS cells were also clearly separated from fibroblasts and overlapped with ES cells. Importantly, regarding the more indicative component 2 (50.73%), the scatter was very low between ES and iPS cells (Fig. 4A). For further analysis, transcripts were assigned to functional groups, and an enrichment test for gene ontology (GO) terms (corresponding to the functional groups) was performed (Fig. 4B). Between the generated iPS cells and the starting material, i.e. primary fibroblasts, 50 GO terms were identified based on statistically significant differential gene expression (Fig. 4B). In contrast, only four GO terms were found to be different between the novel iPS cells and ES cells (with a lower FDR values compared to the iPS cell—fibroblast comparison). These data clearly demonstrate a shift from the somatic fibroblast transcriptome to the “pluripotent transcriptome” reflecting the switch of the cellular program during reprogramming. Interestingly, hierarchical clustering of the three cell types which is based on all detected transcripts, and not only on the ones represented by the principal components shown in Fig. 4A, showed that the iPS cells are even more distant from the fibroblasts than the ES cells are (Fig. 4C).

### Promoter methylation analysis

We tested the methylation status of five gene promoters in the DPZcj_iPSC1 line and included ES cells and fibroblasts as controls. The analyzed promoters were *VASA*, *MAGEA4* (both germ cell-specific genes), *H19* (expressed exclusively from the maternal allele), *MEST* (expression from the paternal allele), and *OCT4* (active only in pluripotent cells and immature germ cells). The comparison between ES cells and fibroblasts showed that *VASA* and *MAGEA4* were highly methylated in both cell types indicating the absence of germ cell gene expression (Fig. 4D). *H19* and *MEST* promoter methylation was around 60% in both cell types. *OCT4* methylation was below 15% in ES cells consistent with strong *OCT4* gene expression in this cell type. In contrast, *OCT4* methylation was around 40% in fibroblasts. *VASA* and *MAGEA4* promoters were highly methylated (80–100%) in all cells tested. In general, the promoter methylation pattern of the iPS cell line DPZcjiPSC1 was very similar to the pattern of the ES cell line (Fig. 4D). These data show that the iPS cells were also reprogrammed on the epigenetic level.

## Discussion

We have derived six novel iPS cell lines from the marmoset monkey using a novel reversible six-factor-in-one-construct *piggyBac* transposon. The iPS cells are pluripotent as shown by direct comparison with ES cells. The cells are stable over time as the first iPS cell line DPZcj_iPSC1 is currently in culture for over 80 passages. The remaining five iPS cell lines were all cultured for at least 35 passages. This shows that marmoset iPS cell generation using the *piggyBac* system is a well-established and reliable procedure. Based on this, it is reasonable to consider that iPS cell-derived differentiated cells or tissues in the future may become a novel therapeutic option for cell and tissue degenerative diseases like myocardial degeneration or neurodegenerative diseases, e.g. Parkinson’s disease. However, such therapies have to be carefully evaluated regarding safety and efficacy. Efficacy testing makes particularly sense in non-human primates (NHP) due to the genetic, immunological, physiological and (neuro-) anatomical similarities between humans and NHP [52]. Safety testing in NHP is also very advantageous compared to other species because of the long life span of the NHP in combination with the above mentioned biological similarities with the human. Altogether, preclinical testing of cell replacement therapies in NHP should generally be more informative than testing in e.g. rodent species. In order to develop a test scenario which is close to the assumed clinical setting, it is necessary to use genetically unmodified stem cells. Transgene-free autologous iPS cells may be particularly useful. Towards this goal, the establishment of robust non-viral *piggyBac*-mediated iPS cell generation is a major step. Consequently, the next steps will be the excision of the *piggyBac* cassette as well as feeder (xeno)-free reprogramming of marmoset cells.

Primate pluripotent stem cells generally have a morphology different from mouse pluripotent stem cells when grown on mouse embryonic feeder cells (reviewed in [53]). While mouse stem cells grow as dome-shaped colonies, primate stem cell colonies are rather flat and have an epitheloid cell layer as top layer of the colonies [54]. Previously described marmoset monkey iPS cell lines were either generated with four [46,47] or six [36] reprogramming factors. Interestingly, the four-factor marmoset monkey iPS cells had unexpected morphologies. The iPS cells reported by Wiedemann and colleagues exhibited morphology close to mouse ES cells, i.e. a rather dome-like shape. The iPS cells reported by Wu and colleagues did not show any colony formation, i.e. the cells did not develop cell clusters characterized by an epitheloid cell morphology, tight cell associations and clearly defined colony boundaries. In contrast, the iPS cells generated in the study by Tomioka and colleagues showed the typical primate pluripotent stem cell colony morphology [36]. This suggests that LIN28 and probably also NANOG are required in addition to the four original Yamanaka factors for full morphological dedifferentiation of marmoset fibroblasts. However, also the marmoset iPS cells generated with four factors were pluripotent as shown by teratoma formation assays, despite of their unexpected morphology. Future comparative gene expression studies will reveal the effects of NANOG and LIN28 during reprogramming of marmoset monkey fibroblasts.

The published studies on marmoset monkey iPS cells used the human factors for reprogramming. Although the reprogramming factors are generally well conserved between human and marmoset, there are some differences between them. Particularly, *NANOG* is less conserved than the other factors (only 88.5% sequence identity on the amino acid level, see S1 Table). Interestingly, Tomioka et al. proved the four classical human Yamanaka factors plus LIN28 to be sufficient for generation of marmoset iPS cells, while reprogramming with the four factors plus NANOG did not succeed. Since we were initially expecting lower reprogramming efficiencies using a non-viral DNA delivery method, we decided to establish a fully homologous system with monkey components only. In order to ensure optimal protein-protein and transcription factor-promoter-interactions, we cloned all six marmoset monkey factors. Hence, the present study is the only one using a homologous system with marmoset monkey reprogramming factors in marmoset monkey cells. This may be of special relevance during the initial phase of reprogramming, which is, before the endogenous pluripotency factors of marmoset origin are activated, driven exclusively by the exogenous factors.

We consider the generated iPS cell lines reprogrammed, although, as expected since under control of the CAG promoter [30,31], the introduced reprogramming cassette is not silenced. Since expression from the reprogramming cassette does not respond to the cells’ gene regulatory mechanisms, the expression from the endogenous loci has to be adjusted to the total amount of protein within the cells. We assume that this adjustment keeps endogenous expression, particular of *SOX2*, moderate as long as the reprogramming cassette is transcribed. Based on the comparison of the expression of the reprogramming cassette and *SOX2* expression levels in the one high-passage number iPS cell line (DPZcj_iPSC1) and the five lower-number iPS cell lines (DPZcj_iPSC2–6; see Fig. 2E), we speculate that the expression of the reprogramming cassette, which is driven by the robust CAG promoter [30,31], is decreased slowly with increasing passage number. The expression level of the reprogramming cassette is lowest in DPZcj_iPSC1 (approximately at passage 80). In contrast, endogenous *SOX2* expression in this cell line is highest when compared to the younger iPS cell lines (passage numbers between 33 and 48). In the younger lines we observed the reverse pattern with higher expression of the reprogramming cassette and relatively low expression of endogenous *SOX2*. We normalized the gene expression levels to a single marmoset ES cell line that was available as reference. By the time of qPCR analysis this line was in a very high passage (248) suggesting that the cells underwent a long selection process for high proliferation rates and pluripotency. In contrast, the passage numbers of iPS cells were 33–84. The extremely long selection process of the ES cells may have influenced the reference level to which the newly generated iPS cell lines were compared. Noteworthy, expression levels of total (endogenous and exogenous) *SOX2*, *OCT4* and *NANOG* were higher in iPSCs than in ES cells (Fig. 2D).

Interestingly, an additional *OCT4* transcript which contains the pluripotency-associated *OCT4A-*specific exon 1 and differs only by two nucleotides from the normal *OCT4A* transcript is annotated in the ENSEMBL database. Discrimination between the two transcripts by PCR, however, would only be possible with TaqMan Assays. Importantly, both isoforms are also present in human cells questioning the significance of some PCR analyses of *OCT4A* expression.

In summary, we have established and applied for the first time robust protocols for the generation of iPS cells from the common marmoset monkey using a novel reversible six factor *piggyBac* construct including selection markers. This NHP species is gaining more and more preclinical relevance due to an increasing number of disease models. Transgene-free marmoset monkey iPS cells will be the basis for the preclinical assessment of “patient-specific” cell replacement therapies using the common marmoset monkey as animal model.

## Supporting Information

S1 ARRIVE Checklist ARRIVE Checklist. (PDF)

S1 Fig Morphology of iPS cell colonies. All six generated iPS cell lines exhibit the typical ES cell colony morphology when grown on mouse embryonic fibroblasts as feeder cells. Bars = 100 μm.
(TIF)

S1 Table Sequence identity between the marmoset and human reprogramming factors SOX2, OCT4, KLF4, c-MYC, LIN28 and NANOG on the amino acid level. (DOCX)

## References

[1] Takahashi K, Yamanaka S (2006) Induction of pluripotent stem cells from mouse embryonic and adult fibroblast cultures by defined factors. Cell 126: 663–676. 16904174 10.1016/j.cell.2006.07.024

[2] Takahashi K, Tanabe K, Ohnuki M, Narita M, Ichisaka T, Tomoda K, et al (2007) Induction of pluripotent stem cells from adult human fibroblasts by defined factors. Cell 131: 861–872. 18035408 10.1016/j.cell.2007.11.019

[3] Yu J, Hu K, Smuga-Otto K, Tian S, Stewart R, Slukvin II, et al (2009) Human induced pluripotent stem cells free of vector and transgene sequences. Science 324: 797–801. 10.1126/science.1172482 19325077 PMC2758053

[4] Okita K, Matsumura Y, Sato Y, Okada A, Morizane A, Okamoto S, et al (2011) A more efficient method to generate integration-free human iPS cells. Nat Methods 8: 409–412. 10.1038/nmeth.1591 21460823

[5] Mack AA, Kroboth S, Rajesh D, Wang WB (2011) Generation of induced pluripotent stem cells from CD34+ cells across blood drawn from multiple donors with non-integrating episomal vectors. PLoS One 6: e27956 10.1371/journal.pone.0027956 22132178 PMC3222670

[6] Fontes A, Macarthur CC, Lieu PT, Vemuri MC (2013) Generation of human-induced pluripotent stem cells (hiPSCs) using episomal vectors on defined Essential 8 Medium conditions. Methods Mol Biol 997: 57–72. 10.1007/978-1-62703-348-0_6 23546748

[7] Stadtfeld M, Nagaya M, Utikal J, Weir G, Hochedlinger K (2008) Induced pluripotent stem cells generated without viral integration. Science 322: 945–949. 10.1126/science.1162494 18818365 PMC3987909

[8] Fusaki N, Ban H, Nishiyama A, Saeki K, Hasegawa M (2009) Efficient induction of transgene-free human pluripotent stem cells using a vector based on Sendai virus, an RNA virus that does not integrate into the host genome. Proc Jpn Acad Ser B Phys Biol Sci 85: 348–362. 19838014 10.2183/pjab.85.348 PMC3621571

[9] Nishishita N, Takenaka C, Fusaki N, Kawamata S (2011) Generation of human induced pluripotent stem cells from cord blood cells. J Stem Cells 6: 101–108. 23264996

[10] Ban H, Nishishita N, Fusaki N, Tabata T, Saeki K, Shikamura M, et al (2011) Efficient generation of transgene-free human induced pluripotent stem cells (iPSCs) by temperature-sensitive Sendai virus vectors. Proc Natl Acad Sci U S A 108: 14234–14239. 10.1073/pnas.1103509108 21821793 PMC3161531

[11] Yang G, Si-Tayeb K, Corbineau S, Vernet R, Gayon R, Dianat N, et al (2013) Integration-deficient lentivectors: an effective strategy to purify and differentiate human embryonic stem cell-derived hepatic progenitors. BMC Biol 11: 86 10.1186/1741-7007-11-86 23870169 PMC3751548

[12] Warren L, Manos PD, Ahfeldt T, Loh YH, Li H, Lau F, et al (2010) Highly efficient reprogramming to pluripotency and directed differentiation of human cells with synthetic modified mRNA. Cell Stem Cell 7: 618–630. 10.1016/j.stem.2010.08.012 20888316 PMC3656821

[13] Warren L, Ni Y, Wang J, Guo X (2012) Feeder-free derivation of human induced pluripotent stem cells with messenger RNA. Sci Rep 2: 657 10.1038/srep00657 22984641 PMC3442198

[14] Warren L, Wang J (2013) Feeder-free reprogramming of human fibroblasts with messenger RNA. Curr Protoc Stem Cell Biol 27: Unit 4A 6 10.1002/9780470151808.sc04a06s27 24510287

[15] Mandal PK, Rossi DJ (2013) Reprogramming human fibroblasts to pluripotency using modified mRNA. Nat Protoc 8: 568–582. 10.1038/nprot.2013.019 23429718

[16] Wang P, Na J (2013) Reprogramming to pluripotency and differentiation of cells with synthetic mRNA. Methods Mol Biol 969: 221–233. 10.1007/978-1-62703-260-5_14 23296937

[17] Zhou H, Wu S, Joo JY, Zhu S, Han DW, Lin T, et al (2009) Generation of induced pluripotent stem cells using recombinant proteins. Cell Stem Cell 4: 381–384. 10.1016/j.stem.2009.04.005 19398399 PMC10182564

[18] Hou P, Li Y, Zhang X, Liu C, Guan J, Li H, et al (2013) Pluripotent stem cells induced from mouse somatic cells by small-molecule compounds. Science 341: 651–654. 10.1126/science.1239278 23868920

[19] Montserrat N, Nivet E, Sancho-Martinez I, Hishida T, Kumar S, Miquel L, et al (2013) Reprogramming of human fibroblasts to pluripotency with lineage specifiers. Cell Stem Cell 13: 341–350. 10.1016/j.stem.2013.06.019 23871606

[20] Shu J, Wu C, Wu Y, Li Z, Shao S, Zhao W, et al (2013) Induction of pluripotency in mouse somatic cells with lineage specifiers. Cell 153: 963–975. 10.1016/j.cell.2013.05.001 23706735 PMC4640445

[21] Anokye-Danso F, Trivedi CM, Juhr D, Gupta M, Cui Z, Tian Y, et al (2011) Highly efficient miRNA-mediated reprogramming of mouse and human somatic cells to pluripotency. Cell Stem Cell 8: 376–388. 10.1016/j.stem.2011.03.001 21474102 PMC3090650

[22] Kaji K, Norrby K, Paca A, Mileikovsky M, Mohseni P, Woltjen K (2009) Virus-free induction of pluripotency and subsequent excision of reprogramming factors. Nature 458: 771–775. 10.1038/nature07864 19252477 PMC2667910

[23] Woltjen K, Michael IP, Mohseni P, Desai R, Mileikovsky M, Hamalainen R, et al (2009) piggyBac transposition reprograms fibroblasts to induced pluripotent stem cells. Nature 458: 766–770. 10.1038/nature07863 19252478 PMC3758996

[24] Wang W, Lin C, Lu D, Ning Z, Cox T, Melvin D, et al (2008) Chromosomal transposition of PiggyBac in mouse embryonic stem cells. Proc Natl Acad Sci U S A 105: 9290–9295. 10.1073/pnas.0801017105 18579772 PMC2440425

[25] Liu G, Aronovich EL, Cui Z, Whitley CB, Hackett PB (2004) Excision of Sleeping Beauty transposons: parameters and applications to gene therapy. J Gene Med 6: 574–583. 15133768 10.1002/jgm.486 PMC1865527

[26] Zeng X, Couture LA (2013) Pluripotent stem cells for Parkinson’s disease: progress and challenges. Stem Cell Res Ther 4: 25 10.1186/scrt173 23672848 PMC3707048

[27] Holditch SJ, Terzic A, Ikeda Y (2014) Concise review: pluripotent stem cell-based regenerative applications for failing beta-cell function. Stem Cells Transl Med 3: 653–661. 10.5966/sctm.2013-0184 24646490 PMC4006488

[28] Chen WW, Blurton-Jones M (2012) Concise review: Can stem cells be used to treat or model Alzheimer’s disease? Stem Cells 30: 2612–2618. 10.1002/stem.1240 22997040 PMC3508338

[29] Kobayashi Y, Okada Y, Itakura G, Iwai H, Nishimura S, Yasuda A, et al (2012) Pre-evaluated safe human iPSC-derived neural stem cells promote functional recovery after spinal cord injury in common marmoset without tumorigenicity. PLoS One 7: e52787 10.1371/journal.pone.0052787 23300777 PMC3531369

[30] Shiozawa S, Kawai K, Okada Y, Tomioka I, Maeda T, Kanda A, et al (2011) Gene targeting and subsequent site-specific transgenesis at the beta-actin (ACTB) locus in common marmoset embryonic stem cells. Stem Cells Dev 20: 1587–1599. 10.1089/scd.2010.0351 21126169

[31] Wistuba J, Luetjens CM, Ehmcke J, Redmann K, Damm OS, Steinhoff A, et al (2013) Experimental endocrine manipulation by contraceptive regimen in the male marmoset (Callithrix jacchus). Reproduction 145: 439–451. 10.1530/REP-12-0373 23431271

[32] Vogt EJ, Meglicki M, Hartung KI, Borsuk E, Behr R (2012) Importance of the pluripotency factor LIN28 in the mammalian nucleolus during early embryonic development. Development 139: 4514–4523. 10.1242/dev.083279 23172912 PMC3912245

[33] Lin ZY, Imamura M, Sano C, Nakajima R, Suzuki T, Yamadera R, et al (2012) Molecular signatures to define spermatogenic cells in common marmoset (Callithrix jacchus). Reproduction 143: 597–609. 10.1530/REP-11-0215 22323619

[34] Okano H, Hikishima K, Iriki A, Sasaki E (2012) The common marmoset as a novel animal model system for biomedical and neuroscience research applications. Semin Fetal Neonatal Med 17: 336–340. 10.1016/j.siny.2012.07.002 22871417

[35] Kishi N, Sato K, Sasaki E, Okano H (2014) Common marmoset as a new model animal for neuroscience research and genome editing technology. Dev Growth Differ 56: 53–62. 10.1111/dgd.12109 24387631

[36] Tomioka I, Maeda T, Shimada H, Kawai K, Okada Y, Igarashi H, et al (2010) Generating induced pluripotent stem cells from common marmoset (Callithrix jacchus) fetal liver cells using defined factors, including Lin28. Genes Cells 15: 959–969. 10.1111/j.1365-2443.2010.01437.x 20670273 PMC2970909

[37] Mueller T, Fleischmann G, Eildermann K, Matz-Rensing K, Horn PA, Sasaki E, et al (2009) A novel embryonic stem cell line derived from the common marmoset monkey (*Callithrix jacchus*) exhibiting germ cell-like characteristics. Hum Reprod 24: 1359–1372. 10.1093/humrep/dep012 19251728

[38] Miyazaki J, Takaki S, Araki K, Tashiro F, Tominaga A, Takatsu K, et al (1989) Expression vector system based on the chicken beta-actin promoter directs efficient production of interleukin-5. Gene 79: 269–277. 2551778 10.1016/0378-1119(89)90209-6

[39] Alexopoulou AN, Couchman JR, Whiteford JR (2008) The CMV early enhancer/chicken beta actin (CAG) promoter can be used to drive transgene expression during the differentiation of murine embryonic stem cells into vascular progenitors. BMC Cell Biol 9: 2 10.1186/1471-2121-9-2 18190688 PMC2254385

[40] Yusa K, Zhou L, Li MA, Bradley A, Craig NL (2011) A hyperactive piggyBac transposase for mammalian applications. Proc Natl Acad Sci U S A 108: 1531–1536. 10.1073/pnas.1008322108 21205896 PMC3029773

[41] Eildermann K, Aeckerle N, Debowski K, Godmann M, Christiansen H, Heistermann M, et al (2012) Developmental expression of the pluripotency factor sal-like protein 4 in the monkey, human and mouse testis: restriction to premeiotic germ cells. Cells Tissues Organs 196: 206–220. 10.1159/000335031 22572102

[42] Ding S, Wu X, Li G, Han M, Zhuang Y, Xu T (2005) Efficient transposition of the piggyBac (PB) transposon in mammalian cells and mice. Cell 122: 473–483. 16096065 10.1016/j.cell.2005.07.013

[43] Donnelly ML, Hughes LE, Luke G, Mendoza H, ten Dam E, Gani D, et al (2001) The ‘cleavage’ activities of foot-and-mouth disease virus 2A site-directed mutants and naturally occurring &rsquo;2A-like’ sequences. J Gen Virol 82: 1027–1041. 11297677 10.1099/0022-1317-82-5-1027

[44] Donnelly ML, Luke G, Mehrotra A, Li X, Hughes LE, Gani D, et al (2001) Analysis of the aphthovirus 2A/2B polyprotein ‘cleavage’ mechanism indicates not a proteolytic reaction, but a novel translational effect: a putative ribosomal ‘skip’. J Gen Virol 82: 1013–1025. 11297676 10.1099/0022-1317-82-5-1013

[45] Szymczak AL, Workman CJ, Wang Y, Vignali KM, Dilioglou S, Vanin EF, et al (2004) Correction of multi-gene deficiency in vivo using a single ‘self-cleaving’ 2A peptide-based retroviral vector. Nat Biotechnol 22: 589–594. 15064769 10.1038/nbt957

[46] Wu Y, Zhang Y, Mishra A, Tardif SD, Hornsby PJ (2010) Generation of induced pluripotent stem cells from newborn marmoset skin fibroblasts. Stem Cell Res 4: 180–188. 10.1016/j.scr.2010.02.003 20363201 PMC2875323

[47] Wiedemann A, Hemmer K, Bernemann I, Gohring G, Pogozhykh O, Figueiredo C, et al (2012) Induced pluripotent stem cells generated from adult bone marrow-derived cells of the nonhuman primate (Callithrix jacchus) using a novel quad-cistronic and excisable lentiviral vector. Cell Reprogram 14: 485–496. 10.1089/cell.2012.0036 23194452

[48] Wislocki GB (1939) Observations on twinning in marmosets. American Journal of Anatomy 64: 445–483.

[49] Benirschke K, Anderson JM, Brownhill LE (1962) Marrow Chimerism in Marmosets. Science 138: 513–515. 17753948 10.1126/science.138.3539.513

[50] Boyer LA, Lee TI, Cole MF, Johnstone SE, Levine SS, Zucker JP, et al (2005) Core transcriptional regulatory circuitry in human embryonic stem cells. Cell 122: 947–956. 16153702 10.1016/j.cell.2005.08.020 PMC3006442

[51] Wickham H (2009) ggplot2: elegant graphics for data analysis: Springer.

[52] Phillips KA, Bales KL, Capitanio JP, Conley A, Czoty PW, t Hart BA, et al (2014) Why primate models matter. Am J Primatol 76: 801–827. 10.1002/ajp.22281 24723482 PMC4145602

[53] Nakatsuji N, Suemori H (2002) Embryonic stem cell lines of nonhuman primates. ScientificWorldJournal 2: 1762–1773. 12806169 10.1100/tsw.2002.829 PMC6009524

[54] Behr R, Heneweer C, Viebahn C, Denker HW, Thie M (2005) Epithelial-mesenchymal transition in colonies of rhesus monkey embryonic stem cells: a model for processes involved in gastrulation. Stem Cells 23: 805–816. 15917476 10.1634/stemcells.2004-0234

