# Supplementary figures and images for: Non-Viral Generation of Marmoset Monkey iPS Cells by a Six-Factor-in-One-Vector Approach
Source: PLoS One. 2015 Mar 18;10(3):e0118424. doi: 10.1371/journal.pone.0118424 (PMC4365012; doi:10.1371/journal.pone.0118424)

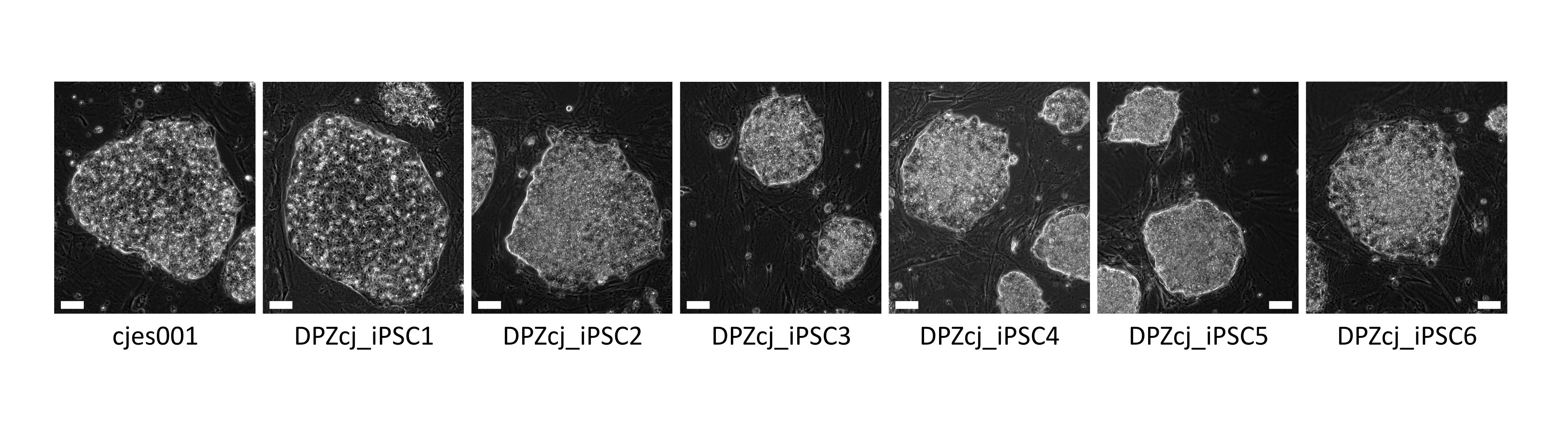

Supplement: S1 Fig — All six generated iPS cell lines exhibit the typical ES cell colony morphology when grown on mouse embryonic fibroblasts as feeder cells. Bars = 100 μm. (TIF) [file pone.0118424.s002.tif]
