# Supplementary material for: Non-Viral Generation of Marmoset Monkey iPS Cells by a Six-Factor-in-One-Vector Approach
Source: PLoS One. 2015 Mar 18;10(3):e0118424. doi: 10.1371/journal.pone.0118424 (PMC4365012; doi:10.1371/journal.pone.0118424)
Supplement: S1 Table — (DOCX) [file pone.0118424.s003.docx]

**Table S2:** Sequence identity between the marmoset and human reprogramming factors SOX2, OCT4, KLF4, cMYC, LIN28 and NANOG on the amino acid level.

| **Protein** | **Identity marmoset vs. human** |
| --- | --- |
| SOX2 | 98.1% |
| OCT4 | 97.5% |
| KLF4 | 97.2% |
| cMYC | 96.1% |
| LIN28 | 100.0% |
| NANOG | 88.5% |
